# Amazing Fungi for Eco-Friendly Composite Materials: A Comprehensive Review

**DOI:** 10.3390/jof8080842

**Published:** 2022-08-11

**Authors:** Worawoot Aiduang, Athip Chanthaluck, Jaturong Kumla, Kritsana Jatuwong, Sirasit Srinuanpan, Tanut Waroonkun, Rawiwan Oranratmanee, Saisamorn Lumyong, Nakarin Suwannarach

**Affiliations:** 1 Department of Biology, Faculty of Science, Chiang Mai University, Chiang Mai 50200, Thailand; 2 Research Center of Microbial Diversity and Sustainable Utilization, Chiang Mai University, Chiang Mai 50200, Thailand; 3 Faculty of Architecture, Chiang Mai University, Chiang Mai 50200, Thailand; 4 Academy of Science, The Royal Society of Thailand, Bangkok 10300, Thailand

**Keywords:** agricultural waste, biomaterial, innovative design, mycelium-based composite, saprobic fungi

## Abstract

The continually expanding use of plastic throughout our world, along with the considerable increase in agricultural productivity, has resulted in a worrying increase in global waste and related environmental problems. The reuse and replacement of plastic with biomaterials, as well as the recycling of agricultural waste, are key components of a strategy to reduce plastic waste. Agricultural waste is characterized as lignocellulosic materials that mainly consist of cellulose, hemicellulose, and lignin. Saprobe fungi are able to convert agricultural waste into nutrients for their own growth and to facilitate the creation of mycelium-based composites (MBC) through bio-fabrication processes. Remarkably, different fungal species, substrates, and pressing and drying methods have resulted in varying chemical, mechanical, physical, and biological properties of the resulting composites that ultimately vary the functional aspects of the finished MBC. Over the last two decades, several innovative designs have produced a variety of MBC that can be applied across a range of industrial uses including in packaging and in the manufacturing of household items, furniture, and building materials that can replace foams, plastics, and wood products. Materials developed from MBC can be considered highly functional materials that offer renewable and biodegradable benefits as promising alternatives. Therefore, a better understanding of the beneficial properties of MBC is crucial for their potential applications in a variety of fields. Here, we have conducted a brief review of the current findings of relevant studies through an overview of recently published literature on MBC production and the physical, mechanical, chemical, and biological properties of these composites for use in innovative architecture, construction, and product designs. The advantages and disadvantages of various applications of mycelium-based materials (MBM) in various fields have been summarized. Finally, patent trends involving the use of MBM as a new and sustainable biomaterial have also been reviewed. The resulting knowledge can be used by researchers to develop and apply MBC in the form of eco-friendly materials in the future.

## 1. Introduction

Plastics are representative of a wide variety of synthetic or semi-synthetic materials that are primarily comprised of polymers. Worldwide plastic production increased from around 1.5 million metric tons per year in 1950 to an estimated 359 million metric tons in 2018 [1]. Plastics are a versatile and convenient synthetic material that is wildly popular and can be used in the manufacturing of many functional products (e.g., food containers, furniture, plastic bags, and toys) that are used by people around the world in their daily lives [2]. However, single-use plastics pose an ever-increasing environmental threat worldwide because their process of degradation takes place over an extremely long period of time. Consequently, the routine practice of discarding plastic has contributed to an increasingly obvious environmental problem [3,4]. The large amount of plastic waste routinely being put into our environment has led to a range of potentially perilous consequences such as unmanageable levels of pollution, the disintegration of our global biodiversity, wasteful energy practices, and a range of economic losses [5]. Moreover, the recovery of plastic waste has remained at a relatively low rate as the majority of plastic waste is either dumped into the ocean, disposed of in landfills, or burned in incinerators, all of which are known to have harmful effects on human health and contribute to the ever-growing amounts of pollution in our air, water, and land [6,7,8,9,10]. In order to address these problems, researchers have now begun to focus on the development of environmentally friendly biodegradable materials that can be employed in the replacement of plastic with natural and ecologically sustainable materials [11,12].

Agricultural waste that is of lignocellulose origin includes waste that is produced through various agricultural activities or processes. Recently, the amount of agricultural waste being generated through a variety of human activities around the world has grown exponentially [13]. The production of agricultural waste is increasing at a rate of 5–10 percent each year on average [14,15]. Global agricultural waste is expected to rise to around 2.2 billion tons annually by the year 2025 [16,17]. Agricultural residue may pose a significant threat to human health due to the environmental pollution it has been associated with if it is not properly managed. Consequently, environmental problems and the harmful effects of agricultural waste are receiving increasing amounts of attention from scientists and researchers. In response to these problems, many research studies have focused on the development of effective strategies and techniques that use agricultural waste in the manufacturing of high-value-added products (e.g., antibiotics, antioxidants, animal feed, biofuels, enzymes, and vitamins) that are generated through various recycling processes. These processes can reduce production costs and reduce the pollution load within the environment [18]. Additionally, some agricultural waste has been considered for use in the production of biomaterials as eco-friendly biodegradable composite materials [19,20,21]. Furthermore, some researchers have focused on using this waste in the production of mycelium-based composites (MBC) obtained from several mushroom genera [22,23,24,25,26,27]. MBC are biodegradable materials obtained from fungal species that use lignocellulosic waste as a substrate for their growth. Bio-fabrication processes can be used as binders to combine the substrate particles together via mycelia networks. MBC have been developed and widely used in a variety of fields including in the construction, packaging, agriculture, and furniture manufacturing industries. Notably, the properties of MBC can vary depending upon a variety of factors [23,24,25,26,27,28,29,30]. Interestingly, MBC have several major advantages over most commonly used synthetic materials. These include their low cost, low density, and their low levels of energy consumption. They are also considered significantly advantageous for their biodegradability, low environmental impact, and low carbon footprint. However, some limitations, such as their low mechanical properties and high-water absorption capabilities, are major concerns that must be addressed if these composites are to be effectively employed in the production of certain structural materials in the future [27,28,29,30,31,32,33,34,35,36,37,38,39,40,41,42,43,44,45,46,47,48,49,50,51,52,53,54,55,56,57,58,59,60,61,62,63,64,65,66,67,68,69]. Therefore, in this study, we have summarized the current findings on the use of agricultural waste as growth substrates and fungal species for the production of MBC. Herein, the biological, chemical, mechanical, and physical properties of MBC have been summarized. Moreover, the innovative designs of MBM and the relevant patent trends have also been summarized.

## 2. Fungal Species and Agricultural Wastes for Mycelium-Based Composite Production

**Figure 1:**
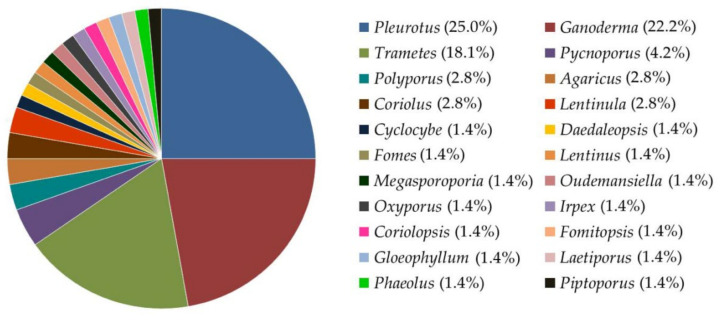
Fungal genera used in mycelium-based composite production.

Fungi, especially saprobic fungi, can be utilized to recycle agricultural waste into biomaterials known as MBC. The common fungal genera, namely *Agaricus*, *Coriolus*, *Coriolopsis*, *Cyclocybe*, *Daedaleopsis*, *Fomes*, *Fomitopsis*, *Ganoderma*, *Gloeophyllum*, *Irpex*, *Laetiporus*, *Lentinula*, *Lentinus*, *Megasporoporia*, *Oudemansiella*, *Oxyporus*, *Pleurotus*, *Phaeolus*, *Piptoporus*, *Polyporus*, *Pycnoporus*, and *Trametes*, that have been classified as white-rot and brown-rot fungi, can be used in the production of MBC due to their high colonization rate and ability to decompose a large amount of organic biomass [22,23,24,25,26,27,28,29,30,31,32,33,34,35,36,37,38,39,40,41,42,43,44,45,46,47,48,49,50,51,52,53,54,55,56,57,58,59,60,61,62,63,64,65,66,67,68,69]. According to the outcomes of previous studies, the genus *Pleurotus* is the most prolific producer of MBC at 25.0%, followed by *Ganoderma* (22.2%), *Trametes* (18.1%), *Pycnoporus* (4.2%), *Polyporus* (2.8%), *Agaricus* (2.8%), *Coriolus* (2.8%), and *Lentinula* (2.8%) (Figure 1). Furthermore, several previous studies have reported that different hyphae network systems can influence the properties of MBC [23,25,69,70]. The three different mycelial network systems can be characterized as monomitic, dimitic, and trimitic, which possess generative, binding, and skeletal hyphae [69,71]. Monomitic systems usually have only generative hyphae, while dimitic systems typically have two types of hyphae (often generative and skeletal), and trimitic systems possess all three hyphal types. According to previous studies conducted by Bayer and McIntyre [72,73], monomitic mycelial network is associated with lower mechanical performance than dimitic and trimitic hyphal systems. For example, *T. versicolor* (trimitic hyphal system) possessed greater mechanical properties (tensile and flexural strength) than *P. ostreatus* (monomitic hyphal system) when grown on rapeseed straw [69]. The degradability and colonization capacities of different fungal species and strains were observed to be impacted by different methods of lignocellulosic enzyme production [74]. Subsequently, many factors, including light, humidity, pH, temperature, and incubation period, are important factors that can affect mycelial growth and colonization on substrates [75].

Agricultural waste is defined as lignocellulosic material due to its major lignocellulosic components that include cellulose, hemicelluloses, and lignin [76]. Generally, cellulose is the most abundant component (35 to 50%) followed by hemicellulose (20 to 35%) and lignin (10 to 25%) [77]. However, the composition of cellulose, hemicellulose, and lignin in agricultural waste varies depending upon the plant species, tissue, and maturity of the plant [76]. Agricultural wastes (e.g., wood chips, sawdust, cotton, flax, hemp, straw, husks, spent mushrooms, sugarcane bagasse, and others) are mainly used as substrates for MBC production due to the fact that they can be degraded by fungi [22,23,24,25,26,27,28,29,30,31,32,33,34,35,36,37,38,39,40,41,42,43,44,45,46,47,48,49,50,51,52,53,54,55,56,57,58,59,60,61,62,63,64,65,66,67,68]. Additionally, agricultural waste has been selected and employed in the production of MBC depending upon the waste that is available in each country. The type of agricultural waste and composition can directly affect mycelium growth because the hyphae are direct contact with the substrate and are known to use essential nutrients garnered from the substrate [57]. Importantly, the addition of various nutrient supplements in the substrates can further support mycelial growth [74].

**Figure 2:**
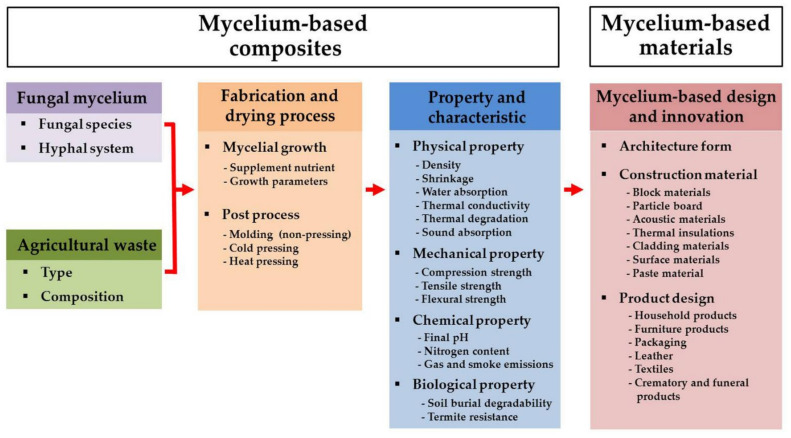
Schematic steps of the synthesis process of mycelium-based composite with key steps and possible variations in processes, and design of mycelium-based materials.

MBC are produced by growing fungal mycelia on different lignocellulosic substrates. Fungi produce mycelia along with a large number of hyphae to form a network on the surface and penetrate the substrate. The hyphae then bind with the substrate particles to form a solid composite [78]. Some fungal mycelia grow out of the substrate and form a compact layer known as the “fungal skin” [79]. Chitin and glucan, which are natural polymers, have been found in mycelial cell walls [80]. Drying processes have been commonly used to stop fungal growth on substrates. Remarkably, differing types of fungal colonization, as well as the different fabrication, drying, and pressing processes being used, have resulted in different properties (chemical, mechanical, and physical) and functional aspects of the resulting MBC [23,81]. Therefore, the selection of the fungal species or strain, the substrate, the fabrication process, and the final finishing process are all crucial considerations in the production of a high-quality MBC (Figure 2).

## 3. Physical Properties of Mycelium-Based Composites

### 3.1. Density

One of the most important physical properties of MBC is its density, which is potentially a key indicator of the beneficial properties of that material. The density of MBC differs according to the substrate type, the fungal species, and the pressing process [52,68]. Differences in density can potentially be related to the differing levels of colonized fungal species that were caused by different lignocellulose-degrading enzyme systems [23]. Lignocellulose degradation causes a change in biomass, which has a direct impact on material density [23,52]. Fungal species and growing substrates are significant factors that influence the density of MBC [23]. For example, *A. bisporus*, *G. lucidum*, and *P. ostreatus* were grown on rapeseed cake to produce MBC with a higher degree of density than composites grown on oat husks [28]. However, MBC produced from *G. lucidum* grown on both rapeseed cakes and oat husks had a lower degree of density than the MBC produced from *A. bisporus* and *P. ostreatus* when grown on the same substrate. Angelova et al. [41] found that the density of the *G. resinaceum* MBC produced on lavender straw was lower than that of rose flower waste. *Pycnoporus sanguineus* grown on pine sawdust produced an MBC with a higher degree of density than coconut powder [51,64]. The obtained density values of various MBC reported in previous studies have been summarized in Table 1. These previous research studies found that MBC possess a density ranging from 25–954 kg/m^3^. Moreover, several other previous studies have determined that the pressing process (cold and/or heated pressing) significantly increased the density of the final MBC [23,25,35,78]. Appels et al. [23] found that heat pressing yielded a 3-fold increase in density, while cold pressing resulted in a 2-fold increase in density when compared with non-pressing for MBC produced from *P. ostreatus* and *T. multicolor* on various substrates. Therefore, controlling the density and homogeneity of MBC has remained a challenge with regard to their potential applications [28,78]. Subsequently, MBC are low in density, normally light weight, and have been associated with a high degree of porosity. Therefore, the density of these MBC has often been compared with the density of certain synthetic foams, e.g., polystyrene, polyurethane, and phenolic formaldehyde resin (11–120 kg/m^3^), and the density of various wood products, e.g., plywood and softwood (440–680 kg/m^3^). It is believed that these composites can potentially be used to replace synthetic foams and products made of plywood and softwood [69].

### 3.2. Shrinkage

Shrinkage value is an important consideration of the physical properties of MBC [82]. The shrinkage of MBC is mostly caused by the dehydration of the samples generated by the drying process [25]. Remarkably, a low shrinkage value can contribute to the strength of the finished product in terms of shape stability. Holt et al. [42] found that the shrinkage value of the MBC made from *Pleurotus* sp. grown on wheat residue was 6.2%, while Elsacker et al. [25] reported that the MBC produced from *T. versicolor* grown on the waste of pine soft wood had a highest shrinkage value (15%) followed by flax (10%) and hemp (9%). This would indicate that the shrinkage value of an MBC can vary depending upon the substrate used. Polymer-based materials (nylon, polystyrene, and polypropylene) were found to have greater shape stability than MBC due to the fact that they are associated with lower shrinkage values (0.3 to 2.5%) [83]. However, the range of shrinkage values reported in prior studies involving MBC was within the range of that reported for wood-based materials (1 to 25%) [84,85]. As a result, they could effectively be used to replace these wood-based materials.

### 3.3. Water Absorption

Typically, MBC are hydroscopic materials [69]. Table 2 summarizes the water absorption ability of various MBC investigated in prior studies. It was found that the water absorption ability varied for different MBC. It can be concluded that the water absorption ability of MBC can vary according to the density of the growing substrate, which typically exhibits high density levels and reduced water absorption ability [28]. Accordingly, the MBC of *G. resinaceum* using rose flower waste displayed significantly lower water absorption ability (43.9%) than lavender straw (114.6%), while rose flower waste had a higher density (462 kg/m^3^) than lavender straw (347 kg/m^3^) [41]. Joshi et al. [58] found that the higher density of sawdust (330 kg/m^3^) when compared with sugarcane bagasse (110 kg/m^3^) resulted in a lower degree of absorption ability in MBC obtained from *P. ostreatus* and sawdust. Moreover, several research studies have concluded that the water absorption ability of a MBC is influenced by the cellulose component, which is usually associated with a large number of accessible hydroxyl groups. This component is characterized by the presence of hydrophobic and hydrophilic mycelia that appear as porous displaying similar absorption times [39,49,52,53,55,58,62,69]. For instance, Appels et al. [23] found that the MBC produced from *T. multicolor* and beech sawdust exhibited lower water absorption ability (43%) than the MBC made from rapeseed straw (436%) at 192 h due to the high density and low content of cellulose of the beech sawdust. Appels et al. [23] and Robertson et al. [86] found that using smaller particle sized substrates can result in fewer voids and pores leading to a higher degree of density and reduced water absorption ability of MBC. Furthermore, Attias et al. [24] found that *T. versicolor* can generate thick hydrophobic mycelia on the surface of the MBC resulting in lower water absorption ability. The standard methods used by the International Organization for Standardization (ISO) and the American Society for Testing and Materials (ASTM) were employed to determine and compare the water absorption values of MBC in relation to other materials (e.g., cement, foam, paper, plastic, and wood). It was found that polymer-based materials (nylon, polystyrene, and polypropylene) possessed lower water absorption abilities (0.01 to 9%) than MBC [69,87]. Remarkably, the high-water absorption problems associated with MBC remain a major challenge in terms of the effective applications of these materials [88]. However, MBC have a variety of uses, including in the insulation and materials used in interior design, whereby the majority of applications are for interior or dry locations that are not exposed to weather, which may help mitigate this critical problem [69].

### 3.4. Thermal Conductivity

Thermal conductivity refers to a material’s ability to transfer or conduct heat. The thermal conductivity of MBC has been investigated and summarized in Table 3. Previous investigations revealed that the thermal conductivity of MBC ranged from 0.029 to 0.104 W/m∙K [22,25,34,37,39,47,56]. Several studies have reported that good insulating materials possess a low level of thermal conductivity [34,37,39,47,68]. Therefore, MBC can possibly be used in the production of certain insulating materials such as conventional commercial thermal insulation products, namely concrete (0.42 W/m∙K), glass wool (0.04 W/m∙K) [89], and polystyrene insulation (0.03 W/m∙K) [90], and other natural insulators including cellulose fiber (0.04 W/m∙K), sheep’s wool (0.05 W/m∙K) [91] and kenaf (0.04 W/m∙K) [92] Notably, the thermal conductivity of MBC was lower than that of cement (1.01 W/m∙K) [89].

### 3.5. Thermal Degradation

The thermal degradation of MBC was investigated over time using thermogravimetric analysis (TGA) at a constant rate of temperature increase [51,59]. The thermal degradation of MBC was similar to the degradation ability that is known to be typical of cellulosic and other biologically derived materials [62]. There are three stages of the thermal degradation process. The first stage involves free and chemically linked water that initially evaporates between 25 °C and 200 °C (5% weight loss). The second stage involves degradation as a much larger mass loss (about 70% weight loss) occurs between 200 to 375 °C, while the last stage involves the decomposition process beginning at 280 to 290 °C [67]. The thermal degradation levels of various MBC reported in previous studies have been summarized in Table 3. The thermal degradation ability of MBC has been observed to be within a range of 225 to 375 °C, as in the range of lignocellulosic materials known to occur between 220 °C and 450 °C [23,51,52,54,59,61,67,93,94]. The thermal degradation of MBC was related to the thermal degradation ability of the lignocellulosic growth substrate, while also being non-affected by the pressing of the fungal species [23]. Additionally, Jones et al. [65] reported that the addition of silica (SiO_2_) in MBC can significantly improve the thermal degradation and fire-resistant capabilities of the composites.

### 3.6. Sound Absorption

Sound absorption is one of the most important factors in the selection and use of sustainable panel and construction materials. Pelletier et al. [44] investigated the sound absorption properties of various MBC (cotton bur fiber, flax shive, hemp pith, kenaf fiber, rice straw, sorghum fiber, and switch grass) using the standard method of ISO10534-1. It was determined that the sound absorption properties of the MBC were within a range of 70–75% absorption at 1000 Hz. MBC can also provide a wider range of sound absorption that is comparable to and can be used in place of other sound absorbers, e.g., fiber boards (11–31%), polystyrene foams (20–60%), polyurethane foams (20–80%), plywood (10–23%), and softwood (5–15%) [45,69]. Pelletier et al. [44] also found that all MBC could reduce the intensity of reflected noise to levels associated with perceptual road noise (45.5–60.0 dBa) and to lower levels than certain reference absorbers, e.g., commercial ceiling tiles (61.0 dBa), plywood (65.0 dBa), and urethane foam boards (64.0 dBa). Moreover, the use of a mixture of substrates in MBC production can result in better sound absorbers than individual substrates. MBC made from mixed rice straw and sorghum fiber (50: 50 mixture ratio) produced the best sound absorber (45.5 dBa) followed by rice straw mixed cotton bur fiber (47.0 dBa) and sorghum fiber mixed switchgrass (47.0 dBa). When compared with individual substrates, rice straw was the best MBC for producing sound absorbers (52.0 dBa) followed by hemp pith (53.0 dBa), flax shive (53.5 dBa), sorghum fiber (54.0 dBa), and switchgrass (55.0 dBa). Furthermore, Castagnede et al. [95] reported that the act of pressing can reduce the sound absorption ability of the finished product. Therefore, it is not recommended to press MBC that are used as sound absorbers through the use of either hot or cold pressing methods.

## 4. Mechanical Properties of Mycelium-Based Composites

### 4.1. Compression Strength

Compressive strength is the ability of a material or structure to withstand loads tending to compress that material and is an important mechanical property that can be used to indicate a key feature in the creation of functional materials [27]. In prior studies, the compressive strength of MBC has been reported to range from 0.03 to 4.44 MPa (Table 4). The compressive strength of MBC varied depending upon the substrate type and the fungi [25,28,35,96]. MBC produced by pine sawdust and *Py. sanguineus* exhibited higher compression strength than *P. albidus* [51]. MBC produced by *G. lucidum* grown on both rapeseed cakes and oat husks had a higher compression strength value than MBC produced from *A. bisporus* and *P. ostreatus* when grown on the same substrate [28]. The MBC of *G. resinaceum* produced on lavender straw (0.72 MPa) has lower compression strength than that of rose flower waste (1.03 MPa) [41]. Additionally, Ghazvinian et al. [26] a reported that the compressive strength of MBC produced from *P. ostreatus* grown on sawdust (1.02 MPa) was significantly higher than when straw was used (0.07 MPa). *Trametes versicolor* grown on hemp produce an MBC with higher compression strength than pine wood and flax [25]. Moreover, the application of pressing during the fabrication process increased the compressive strength in these MBC [35,97]. Currently, the compressive strength of MBC used in the creation of packaging and construction materials is still a major concern. This is because there are a number of problems associated with materials that possess low compressive strength [88]. Thus, the improved compressive strength of MBC in the development of packaging and construction materials is still of critical importance.

### 4.2. Tensile Strength

Tensile strength is one of the most remarkable properties of MBC. The tensile strength of the MBC in previous studies was within the range of 0.01 to 1.55 MPa (Table 4). Several studies found that the tensile strength of MBC can be influenced by the structure of the mycelium binder network which varies depending upon the type of mycelium network [23,69,70,72,73]. *Trametes multicolor* (trimitic hyphal system) on rapeseed straw was employed to create an MBC with higher tensile strength (0.04 MPa) than *P. ostreatus* (monomitic hyphal system) on rapeseed straw (0.01 MPa) because trimitic hyphal systems are more complex than monomitic hyphal systems [23]. The binding and skeletal hyphae in the trimitic hyphal network are characterized by high branching, an interwoven appearance, and the presence of thick cell walls that contribute to the stiffness of the MBC [23,70]. Moreover, the pressing technique could result in the improved tensile properties of MBC [35,38,70]. According to the findings of a study conducted by Appels et al. [23], heat-pressing resulted in the highest degree of tensile strength of the MBC followed by cold-pressing and non-pressing (Table 4). Notably, the previously reported tensile strength of the MBC was similar to that of polystyrene foam (0.15–0.7 MPa) [69].

### 4.3. Flexural Strength

Flexural strength relates to the stress at the fracture points of a sample product when bending [70]. Flexural strength is an important mechanical criterion to consider when employing MBC. The flexural strength of MBC has been summarized in Table 4. It was found that the flexural strength of MBC ranged from 0.05 to 4.40 MPa [23,35,36,54,55]. Appels et al. [23] suggested that the flexural strength of MBC was dependent upon the type of mycelia network and the pressing method used. *Trametes multicolor* (trimitic hyphal system) on rapeseed straw created an MBC with higher flexural strength (0.22 MPa) than *P. ostreatus* (monomitic hyphal system) grown on rapeseed straw (0.06 MPa). Moreover, heat-pressing had a positive correlation with the increased flexural strength of the MBC of *P. ostreatus* grown on rapeseed straw and cotton waste, and that of *T. multicolor* grown on rapeseed straw. The heat-pressing effect resulted in contrasts in the mechanics between the substrate and the fungal mycelium along with the increased elasticity of the MBC [69,70]. The previously determined flexural strength of the MBC was within the range of polystyrene foam (0.07–0.70 MPa) and phenolic formaldehyde resin (0.38–0.78 MPa) [35,36,69]. However, the flexural strength of wood products (8.0–78.0 MPa) was higher than that of the MBC. Thus, MBC may not be suitable for use in the structural applications typically attributed to wood [69].

## 5. Chemical Properties of Mycelium-Based Composites

### 5.1. pH and Nitrogen Content

The chemical properties (e.g., pH, nitrogen content, and organic matter digestion) of MBC have been determined. Several prior studies have reported that the finished MBC had a lower pH value than the starting MBC [24,30,97]. The obtained pH value of the finished MBC ranged from 4.3 to 6.5 (Table 5). Additionally, the amount of nitrogen in the finished MBC was also determined to be a significant chemical property. The obtained values for nitrogen content in the finished MBC in previous studies are summarized in Table 5. The nitrogen content of the finished MBC ranged between 0.5 and 1.6% [30,49,66]. Attias et al. [24,30] found that the nitrogen content was higher than that of the control (non-colonized substrate) and the starting MBC by 1.0–1.7-fold increases. Decreased pH values and increased nitrogen contents are generally caused by enzymatic digestion [97,98]. Therefore, changes in pH value and nitrogen content can be used to assess mycelium colonization and developmental potential [49]. Moreover, Attias et al. [24,30] found that mycelium colonization on a given substrate is associated with the reduced amounts of organic matter in that substrate.

### 5.2. Gas and Smoke Emissions

When an MBC burns, toxic gas emissions, particularly carbon monoxide (CO) and carbon dioxide (CO_2_), represent the greatest danger to human health. Rice hull-based mycelium composite of *T. versicolor* emitted 0.02 g of CO, which was a lower amount of CO than from particleboard made from wood products (0.47 g) and polystyrene foam (0.48 g) and was associated with lower CO_2_ emissions (14.6 g) when compared to polystyrene foam (15.2 g) and particleboard (30.0 g) [69]. Similarly, the wheat grain-based mycelium composite (0.33 g) exhibited CO release behavior that was lower than that of polystyrene foam and particleboard. However, CO_2_ emissions from the wheat grain-based mycelium composite (23.8 g) were higher than that of polystyrene foam. Notably, mycelium composites made from wheat grain and rice hull released smoke at amounts of 70 and 40 m^2^/m^2^, respectively, which were less than polystyrene foam (1184 m^2^/m^2^). MBC made from wheat grain released slightly greater amounts of smoke than particleboard (64 m^2^/m^2^). It can be concluded that MBC emit lower amounts of CO and CO_2_ than polystyrene foam. However, a greater understanding of the capacity of other MBC to emit gas and smoke would require further research. Based on the fact that MBC can generate smoke and harmful gasses when burned, previous studies have determined that incineration is not a preferable waste disposal technique for MBC [27,51,59].

## 6. Biological Properties of Mycelium-Based Composites

### 6.1. Soil Burial Degradability

MBC possess unique properties that include low-cost, safe, biodegradable, and eco-friendly characteristics [70,78,97]. Despite the fact that MBC typically contain natural fibers, the biodegradability of these materials is still unknown due to the absence of standardized testing protocols. Wylick et al. [99] modified a soil burial test for ISO 20200 under laboratory conditions in order to evaluate the degradability of MBC. MBC produced from *T. versicolor* and *G. resinaceum* grown on beech wood and hemp were embedded in the soil, while the weight loss of these MBC was then measured. Weight loss of the MBC increased during 16 weeks of incubation with final weight loss values within the range of 19.06–43.03%. In addition, the degradation of MBC of *T. versicolor* grown on hardwood chips and hemp resulted in a weight loss of over 70% after 12 weeks under conditions of composting [66]. However, the rate of disintegration of MBC was influenced by various parameters [99,100]. Importantly, other aspects of MBC degradation, including material composition, pressing, physical and chemical characteristics, microorganisms (bacteria and fungi), and weathering resilience, have all been undocumented.

### 6.2. Termite Resistance

Termites are highly effective at degrading lignocelluloses and are a serious hazard to residential and commercial buildings in many countries around the world. This would be especially true in Africa, Asia, Australia, and South America [69]. Generally, MBC have no termite resistant properties of their own due to the fact that they are predominantly composed of biological and lignocellulosic materials. However, the termite resistance of MBC can be improved by substrate selection and the application of natural or commercial termiticides. A previous study conducted by Bajwa et al. [31] found that hemp-based mycelium composites exhibited the highest degree of termite-resistance with low mass losses (16–53 wt%), followed by Kenaf-based mycelium composites (43–62 wt%) and corn-based mycelium composites (42–43 wt%) after being exposed to termite infestation over a period of four weeks. The coating of cedar oil, guayule resins, and vetiver oil on MBC resulted in mass losses as 20–32 wt%, 18–28 wt%, and 16–27 wt%, respectively, which were lower than the mass losses of the uncoated MBC (42–62 wt%) and those that were coated with commercial borax termiticide (28–40 wt%). In addition, termite repellence and the mass loss of MBC made from *D. confragosa*, *G. resinaceum*, and *T. versicolor* were not found to be significantly different.

## 7. Critical Assessment of Mycelium-Based Composites

**Figure 3:**
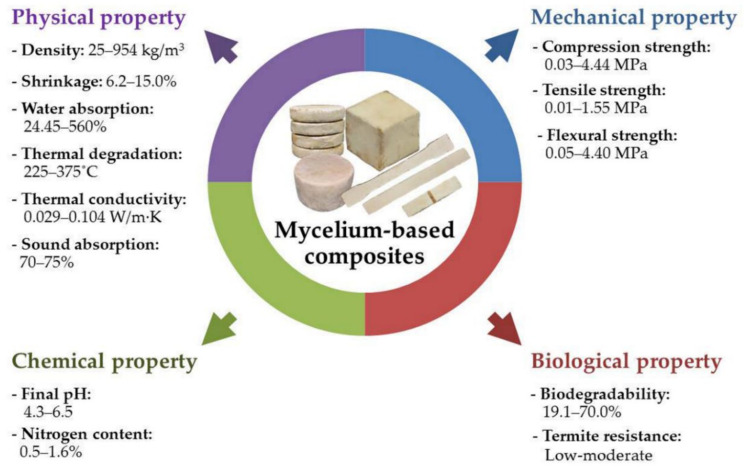
The summarization of physical, mechanical, chemical, and biological properties of the finished mycelium-based composites.

The physical, mechanical, chemical, and biological properties of the finished MBC are critical in the assessment of their potential to be employed in various functional applications (Figure 3). The comparison of physical, mechanical, chemical, and biological properties of MBC with synthetic foams and wood products is shown in Table 6. Low density MBC, such as hemp hurds (98.4 kg/m^3^), oat husks (25–38 kg/m^3^), rapeseed cakes (41–58 kg/m^3^), and sugarcane (98.4 kg/m^3^), can compete with common synthetic foams such as phenolic formaldehyde resin foams (PFR, 35–120 kg/m^3^), polystyrene (PS, 11–50 kg/m^3^), and polyurethane (PU, 30–100 kg/m^3^) [101,102]. After cold or heat pressing, MBC exhibit higher density characteristics (98.4–954 kg/m^3^) than typical synthetic foams, but their degree of density was lower or similar to the degrees of density of hardwood (HW, 850–1030 kg/m^3^), plywood (PW, 460–680 kg/m^3^), and softwood (SW, 440–600 kg/m^3^) [101,102].

Traditional synthetic insulation materials made from polystyrene and polyurethane foams are extremely flammable [69]. On the other hand, MBC offer a major benefit in terms of fire safety when compared to synthetic insulation foams. The fire resistant property of MBC can be improved by the addition of silica [65]. In acoustic insulation applications, MBC offer greater advantages over synthetic foams and wood products. MBC exhibit lower thermal conductivity (0.029–0.104 W/m·K) [22,25,34,37,39,47,56] making them better thermal insulators than hardwood (0.2–0.5 W/m·K), plywood (0.3–0.5 W/m·K), and softwood (0.08–0.3 W/m·K) [101] (Table 6). Moreover, the low thermal conductivity (0.04 W/m·K) of MBC made from hemp fibers or wheat straws can compete with polystyrene (0.03–0.04 W/m·K) [101], polyurethane foam (0.006–0.8 W/m·K) [101,102], and phenolic formaldehyde resin (0.03–0.04 W/m·K) [101,102,103,104,105,106,107] foams. Despite a lack of accumulated data on the noise reduction coefficient (NRC) of MBC, they have been found to provide 70–75% sound absorption at 1000 Hz, which, despite not being a parameter comparable with NRC, suggests that MBC are likely to be competitive with polystyrene foams (20–60% sound absorption, NRC of 0.2–0.6) and polyurethane foams (20–80% sound absorption, NRC of 0.2–0.8). These sound absorption properties are also likely to outperform plywood (10–23% sound absorption, NRC of 0.1–0.23) (Table 6).

**Table 6:** Comparison of properties of mycelium-based composites with synthetic foams and wood products (modified from Jones et al. [69]).

Properties	MBC	Products *						
		Synthetic Foams				Wood Products		
		PS	PU	PFR	PP	PW	SW	HW
Density (kg/m^3^)	25–954	11–50	30–100	35–120	895–920	460–680	440–600	850–1030
Shrinkage (%)	6.2–15.0	0.2–0.6	–	–	1.0–2.5	1–25	6.8–13.8	10.2–19.2
Water absorption (%)	24.45–560	0.03–9	0.01–72	1–15	0.01–0.03	5–49	5–190	5–190
Thermal conductivity (W/m∙K)	0.029–0.104	0.03–0.04	0.006–0.8	0.03–0.04	0.10–0.22	0.3–0.5	0.08–0.3	0.2–0.5
Thermal degradation (°C)	225–375	318–440	278–379	270–475	360–460	250–380	150–276	200–267
Acoustic absorption (%)	70–75	20–60	20–80	–	5–32	10–23	5–15	5–15
Compression strength (MPa)	0.03–4.44	0.03–0.69	0.002–48	0.2–0.55	31.19–48.29	8–25	35–43	68–83
Tensile strength (MPa)	0.01–1.55	0.15–0.7	0.08–103	0.19–0.46	31–41.4	10–44	60–100	132–162
Flexural strength (MPa)	0.05–4.40	0.07–0.70	0.21–57	0.38–0.78	22–23.2	35–78	9.9–11.5	10.3–11.5
Termite resistance	Low-moderate	Low, vulnerable to nesting				Low, excluding heartwood or treated wood		
Final pH	4.3–6.5	–				Wood constituents		
Nitrogen content (%)	0.5–1.6	–				Wood constituents		
Biodegradability (%)	19.1–70.0	–				Wood constituents		

MBC = Mycelium-based composite, PS = polystyrene, PU = polyurethane, PFR = phenolic formaldehyde resin foam, PP = polypropylene, PW = plywood, SW = softwood, HW = hardwood and “–” = not reported. * Bruscato et al. [51], Dizon [83], Forest Products Laboratory [84], Schroeder [85], Ashby [101], MatWeb LLC. [102], Azahari et al. [103], Filip et al. [104], NPCS Board of Consultants & Engineers [105], Niu and Wang [106], Jalalian et al. [107], Papadopoulou and Chrissafis [108], Tailor et al. [109], Deng et al. [110], Dou and Rodrigue [111], Zhu et al. [112], Shen et al. [113], Castro et al. [114], Handayani et al. [115], Goulart et al. [116], Del Menezzi [117], Çolakoğlu and Colak [118], Jivkov et al. [119], Sinha et al. [120], Jamalirad et al. [121], Engineering Toolbox [122], Fateh [123], Zabihzadeh [124], Bodîrlău et al. [125] and Szubel et al. [126].

MBC also possess similar levels of tensile (0.01–1.55 MPa), compressive (0.13–4.44 MPa), and flexural (0.05–4.40 MPa) strength when compared to polyurethane (0.08–103 MPa, 0.002–48 MPa and 0.21–57 MPa, respectively) [101,102] and phenolic formaldehyde resin (0.19–0.46 MPa, 0.2–0.55 MPa and 0.38–0.78 MPa, respectively) foams [101,102,107], but are stronger than polystyrene foams (0.15–0.7 MPa, 0.03–0.69 MPa and 0.07–0.70 MPa, respectively) [101,102]. However, MBC cannot be used in any structural applications traditionally achieved using wood products due to their low tensile, compressive, and flexural strength (Table 6). However, they would very likely be better suited for use in other applications such as in the construction of the cores and panels of doors.

The main disadvantage of mycelium composites is their high-water absorption tendency (24.45–560.00 wt%), which is much higher than polystyrene (0.03–9.00 wt%), polyurethane (0.01–72.00 wt%), and phenolic formaldehyde resin (1–15 wt%) foams and could be a serious problem resulting in leaking walls or roof cavities. Plywood, which has a high moisture uptake capability (5–49 wt%), absorbs less water than MBC, despite the fact that conventional wood is associated with high water absorption properties (5–190 wt%) and exhibits substantial shape changes such as warping. In terms of termite resistance, MBC are similar to untreated wood products and do not offer considerable termite resistance, which could be an issue in regions where termites are a considerable problem. However, synthetic polystyrene foam is susceptible to termite damage with termites occasionally constructing nests within this foam.

Another significant disadvantage of MBC over synthetic foams and wood products is their very slow manufacturing process. It can take days to months to complete this manufacturing process versus the process for synthetic foams and wood products that can be produced in minutes to days depending upon the manufacturing and curing processes [69]. However, the environmental benefits of MBC are that they are fully biodegradable and can simply be composted in soil over the course of a few months at the end of their life. In comparison, wood products are also considered environmentally friendly. Synthetic foams take decades to centuries to decompose in the natural environment, while they are also difficult to recycle and are known to pollute the environment when burned [69].

## 8. Mycelium-Based Designs

Mycelium-based designs have been the subject of interest for more than two decades [127,128]. Early applications began by growing fungal mycelium and agricultural waste on simple molds. Innovative design initiatives and experiments have led to the production of diverse forms of mycelium products that range from packaging to household items, as well as those used in furniture and building materials [26,30]. More complex mycelium technological advancements combining bio-based experiments with bioplastic and other biotech inventions have resulted in the generation of mycelium-based leather and textiles that can be used in clothing and apparel, while continuing innovations have expanded to cover the production of other beneficial products that include food, cosmetics, and medicines [81]. On the other hand, mycelium architecture, also known as fungal architecture and mushroom architecture, has largely been developed over the past ten years with promising potential for broader expansion across the world [129].

### 8.1. Architecture Form

**Figure 4:**
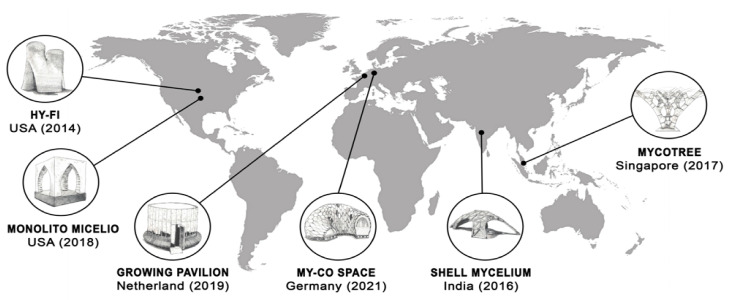
Geographic distribution and year of mycelium-based architecture inventions.

Around the world, mycelium-based architecture has been employed in America, Europe, and Asia. Basically, mycelium-based architecture or fungal architecture evolved from using mycelium-based bricks and panels. Almpani-Lekka et al. [129] have recently reviewed six mycelium-based forms of architecture that include the HyFi Tower, Shell Mycelium Pavilion, Mycotree, Monolito Micelio, Growing Pavilion, and MY-Co Space (Table 7 and Figure 4).

The materials used in these examples of mycelium-based architecture are in their early stage of development. From 2014 until now, these materials have been developed for both outdoor and indoor exhibitions. The buildings are situated on mycelium composite blocks and panels that are supported by bamboo, wood, and/or steel frames and joints. They more or less appear in the form of towers, pavilions, and shelters. The building of a habitable house with built-in domestic functions that employ the principles of mycelium-based design is still underway. Examples of mycelium architecture are most commonly found in America, Asia, and Europe, while the temporal development period has lasted for less than a decade. Therefore, the construction of promising architectural formations will be needed in order to realize the full potential of mycelium-based structures as a habitable form of architecture going forward.

### 8.2. Mycelium Based Construction Materials

**Figure 5:**
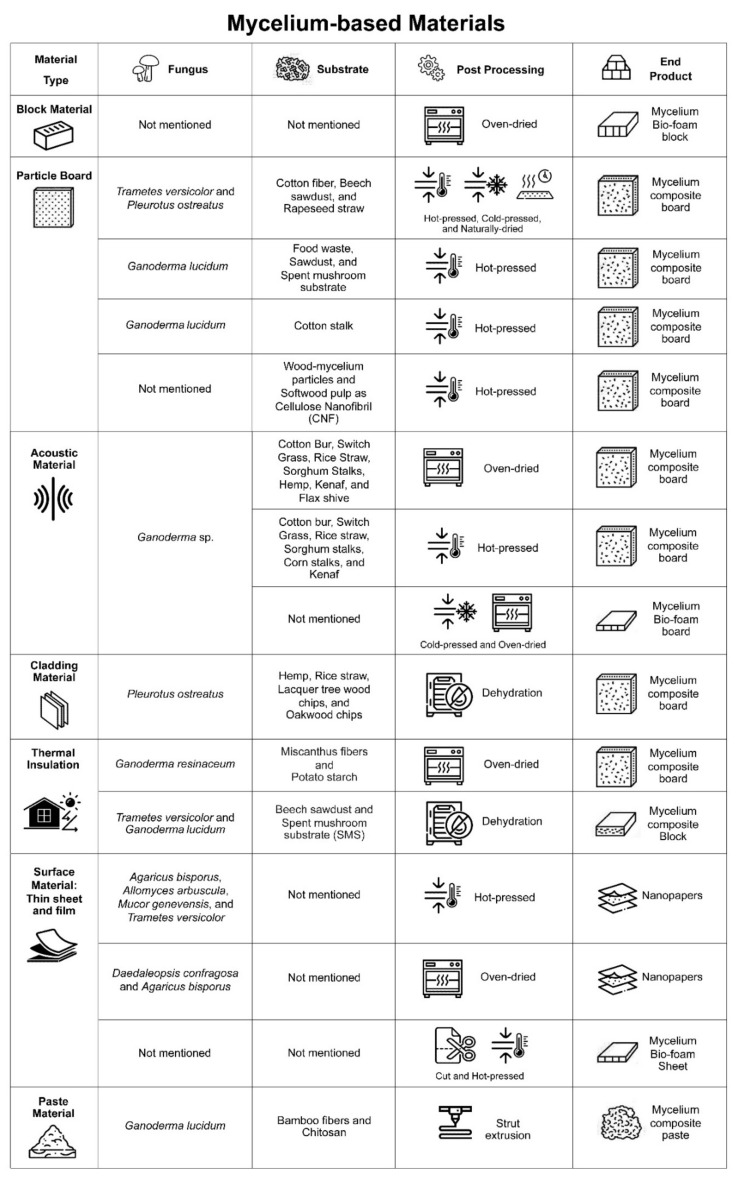
Comparison of mycelium-based material research (Appels et al. [23], Schritt et al. [34], Travaglini et al. [39], Pelletier et al. [44], Pelletier et al. [45], Lee et al. [53], Jones et al. [63], Islam et al. [130], Khoo et al. [131], Liu et al. [132], Sun et al. [133], Pelletier et al. [134], Nawawi et al. [135], Soh et al. [136], and Sun et al. [137]).

A review of mycelium-based construction materials has revealed a considerable amount of progress over the past decade. Cumulative forms of mycelium-based construction materials include block materials [130], particle board [23,131,132,133], acoustic materials [44,45,134], thermal insulations [34,39], cladding materials [53], surface materials (thin sheet and film) [63,133,135] and paste material [136] (Figure 5).

Block materials are material composites exhibiting foam-like characteristics. Islam et al. [130] studied the material response of novel mycelium bio-foam on the multiscale Stochastic Continuum under cyclic compression. This method could be used to generalize predictions on the complex three-dimensional deformation of materials when consideration of density fluctuation and network-like microstructure deem it necessary to use a less complex mesoscale model to design mycelium-based products with the desired scope of mechanical performance for a range of applications. The fiber network employed in the micro-scale model helped to establish heterogeneity in mycelium bio-foam that provides spatial variability in terms of density and non-linear mechanical behavior.

Particle board is developed from a mycelium block through a hot-press process. It is then fabricated into a thinner material and shifts its characteristics from foam-like to more cork-like and wood-like characteristics that can be used in various non-structural applications. The varying degrees of thickness have been researched in several studies [23,131,132]. Appels et al. [23] studied the characteristics of an agricultural-by-product mycelium composite comprised of two types of fungus, including *P. ostreatus* and *T. multicolor*, through the employment of three fabrication processes (no-press, cold press, and hot-press processes). Improvements in the stiffness and homogeneity of the composite were observed after utilizing the hot-press process. The process shifts the elastic modulus of the composite from a foam-like substance to a cork-like and wood-like substance that has a density of 100–390 kg/m^3^. As a result, the weights of both fungus mycelium composites were lighter than those of the medium-density fiberboard (MDF; 500–1000 kg/m^3^) and oriented strand board (OSB; 550–700 kg/m^3^), while the composite still retained the preferred mechanical properties of a conventional wood composite. Khoo et al. [131] investigated mycelium bonding as a natural adhesive in the development of high-strength bio-boards prepared from compressed spent mushroom substrates (SMS) in conjunction with various fungal species. These can minimize the harm associated with formaldehyde-based adhesives that are believed to have hazardous health and environmental effects. Under a temperature of 160 °C and 10 MPa compression force for 20 min, the bio-board exhibited much higher internal bonding strength at up to 2.51 MPa, which is above both the established China and US standard ranges for plywood boards. This bio-board also exhibited other potentially beneficial characteristics in terms of water and fire resistance. Liu et al. [132] investigated the effects of varying temperatures on a novel cotton stalk-mycelium composite that was produced using the hot-press process. With increasing temperatures under 200 °C employed in the hot-press process, new chemical bonds between mycelium and cotton stalk particles occurred to yield a composite structure more compact and displaying improved flexural and internal bonding strength that were comparable to fiberboard. A decrease in thermal decomposition resistance was also observed. Sun et al. [133] developed a mycelium- modified wood panel from softwood particles and fungal mycelium with cellulose nanofibrils (CNF) being used as a natural binder. The addition of CNF improved the physical and mechanical properties of the composites as loading values increased by 5% for 400 kg/m^3^ when CNF was added and 2.5% for 600 kg/m^3^ when CNF was added by forming a network of fungal hyphae. Notably, CNF formed a uniform mycelial film covering the wood particles at a microscopic level. Mycelium modification also reduced water absorption and thickness in the swelling of the composite, while in turn increasing the modulus of rupture and elasticity. The CNF added composite could potentially replace formaldehyde-based lightweight composites by offering optimized physical and mechanical properties and better dimensional stability.

Pelletier et al. [44,45,135] have studied the acoustic applications of renewable fungal material grown on various agricultural by-product substrates since 2013. In conjunction with the impedance tube method, densification via the compression technique on mycelium-based boards was used to determine sound shielding and sound absorption performance. Accordingly, the resulting material could be used as a sustainable alternative to conventional lightweight materials such as MDF and OSB. Moreover, a study of the fruiting body of fungal mycelium in a controlled environment chamber with a combination of elevated temperatures and carbon dioxide levels yielded pure mycelium foam with a closed-cell structure that could alternatively substitute synthetic foam insulation board [45].

Importantly, the density of the material determines its thermal conductivity, which is considered fairly low in good insulation. Dias et al. [39] investigated the suitable mixture proportions of the substrate and mycelium to produce a lower density porous composite. Apart from lower thermal conductivity, the fire resistance properties of the developed composite plates could be elevated and categorized in the EI15 category according to EN13501–2:2003 making them an effective, non-flammable, bio-composite form of material insulation that could reduce the environmental footprint of buildings. Schritt et al. [34] studied the development of a competitive and sustainable lightweight mycelium-based insulation material obtained from recycled beech sawdust (SD) and spent mushroom substrate (SMS) that exhibited properties of low thermal conductivity. *Trametes versicolor* mycelium grown on SD displayed good growth rate (7.4–11.8 mm/day) and handling properties that were associated with mycelial density within the range of 190–200 kg/m^3^. Thus, this process could effectively recycle SMS and *G. lucidum* SD-based substrates into lightweight materials with low thermal conductivity properties (0.06–0.07 W/m·K). In this regard, *G. lucidum* was found to be unstable in the recycling of SMS, while *T. versicolor* effectively utilized SD and SMS by further expanding its recycling options in the production of thermal insulation composites with even lower properties of thermal conductivity.

Cladding material development primarily focuses on surface properties. Lee and Choi [53] investigated the potential utilization of mycelium composites grown on different substrates to be further developed as adsorbing atmospheric particulate matter panels that could be applied to architectural façades. In conjunction with the porous filter-like characteristics of the composite structure, these composites could display better adsorption capabilities than conventional cladding material, while the adsorption performance could be varied depending upon the type of substrate used.

MBC can be fabricated into slightly thin materials, such as thin sheets and films that can then be applied to a range of other products. Jones et al. [63] studied the characterized polymer extracts and nanopapers produced from a common mushroom reference species and various species of fungal mycelium grown on liquid sugarcane by-product molasses. This modified extract heightened the tensile strength of the nanopapers, which exhibited better hydrophobic surface properties that could potentially support their use in a wide range of applications including in coatings, membranes, packaging, and paper. Sun et al. [137] published a report on a ‘Smart mycelium surface’ that possessed a tunable wettability surface that would be beneficial in the production of commercial mycelium foam. This would allow it to maintain its hydrophobicity and non-absorbent characteristics under a temperature of 50 °C. Notably, any relative humidity could turn the surface back to its hydrophobic state and partially restore non-absorbance with a switchable character. Nawawi et al. [135] investigated the mechanical properties of mycelium nanopapers influenced by variations in fiber diameter and chitin to *β*-glucan ratio on a species to species basis. It was determined that the hot-press and mild alkaline processes changed the nanocomposite architecture from brittle and plastic-like to a very tough and elastomeric rubber-like state exhibiting very high tensile strength. Thus, it has been identified as a potential coating agent for hydrophilic materials. These remarkable and controllable characteristics make fungi-derived materials versatile for a wide range of applications including in the production of coatings, membranes, packaging, and paper.

With regard to paste material, Soh et al. [136] studied new extrusion molding techniques for mycelium composite fabrication that currently have limited capacity to be employed in the design of 3D complex shapes. The composition of mycelium obtained from *G. lucidum* in growing with agricultural waste, bamboo fiber, and chitosan could create a workable and extrudable paste-like mixture. The impact of bamboo fiber size (500 μm), chitosan concentration (3 wt%), pH (~6), and the weight ratio of bamboo to chitosan (60: 40) could be used to establish the optimum growth conditions for mycelium. Despite the fact that the use of chitosan decreases the stiffness of the end-product when compared to other products made without chitosan, the composition does offer greater potential to be used in the fabrication of complex shapes of mycelium-bound materials for use in advanced structural applications, such as those that involve 3D printing technology. Along with a widening range of applications for these materials, there are also benefits associated with sustainability and reduced energy costs.

The developmental trends for MBM have mainly focused on applications in composite boards or panels using various agricultural waste and fungi, for which testing has been implemented for their potential applications in both thermal and acoustic insulation. Accordingly, certain heating methods, such the hot-pressing and oven-dry methods, have been employed in the processing of products to create a high-performance, sustainable alternative to conventional wood or foam-based boards. Furthermore, mycelium thin sheets and films that have been developed from various fungi grown on organic substances have also used the heat method to terminate the growth and shape of the mycelium product into thin sheets, films, and nanopapers. These innovative products could replace the polymer-based coating materials that are presently being used in many applications, especially in the area of product design and packaging.

### 8.3. Product Design

Mycelium-based product design employs the molding techniques of basic MBC bricks and results in even greater innovative eco-friendly product design for enhanced commercialized purposes [24,30,36].

#### 8.3.1. Household and Furniture Products

The household products made from combining mycelium and bioplastic technology include a range of household and decorative items such as vases, pots, cups, and lamps [138]. The successful use of mycelium composites in timber frame furniture and other accessories, including lamps, chairs, and tables, is contributing to the generation of a range of increasingly popular design items [139].

#### 8.3.2. Packaging Products

Mycelium based packaging is a rapidly growing area of product line development that further expands the potential of mycelium industrial design products. The leading company in the market is Evocative Design, which offers a range of packaging products made from MBC. These include MycoComposite and a type of mycelium foam being sold as MycoFlex [140]. Another mycelium packaging innovator is Grown, the creator of Growing Pavillion [141].

#### 8.3.3. Leather and Textiles

Advancements in mycelium-based leather and textiles have led to new clothing and apparel product lines that involve mycelium technology. These include, for example, MycoTex [142], MyCoTech [143], Fine Mycelium [144], and Air Mycelium. Production has expanded beyond the manufacturing of leather and textiles. It now includes the development of new forms of artificial meat such as MyBacon [140].

#### 8.3.4. Crematory and Funeral Products

One of the most cutting-edge mycelium-based products is the mycelium-based coffin named the Living Cocoon. This product claims that it can degrade along with a dead body once they are buried in the ground (www.loop-of-life.com (accessed on 1 May 2022)). Further product designs have applied the concept of mycoremeditation or the use of fungi and mushrooms in the removal of waste from the environment [145,146,147]. Furthermore, an innovative project called “Infinity Burial Project” has resulted in the invention of two new products that employ an alternative form of leather used in the burial of dead bodies. These innovative products are now being marketed as a burial suit and a burial shroud [148].

## 9. Patent Search

**Figure 6:**
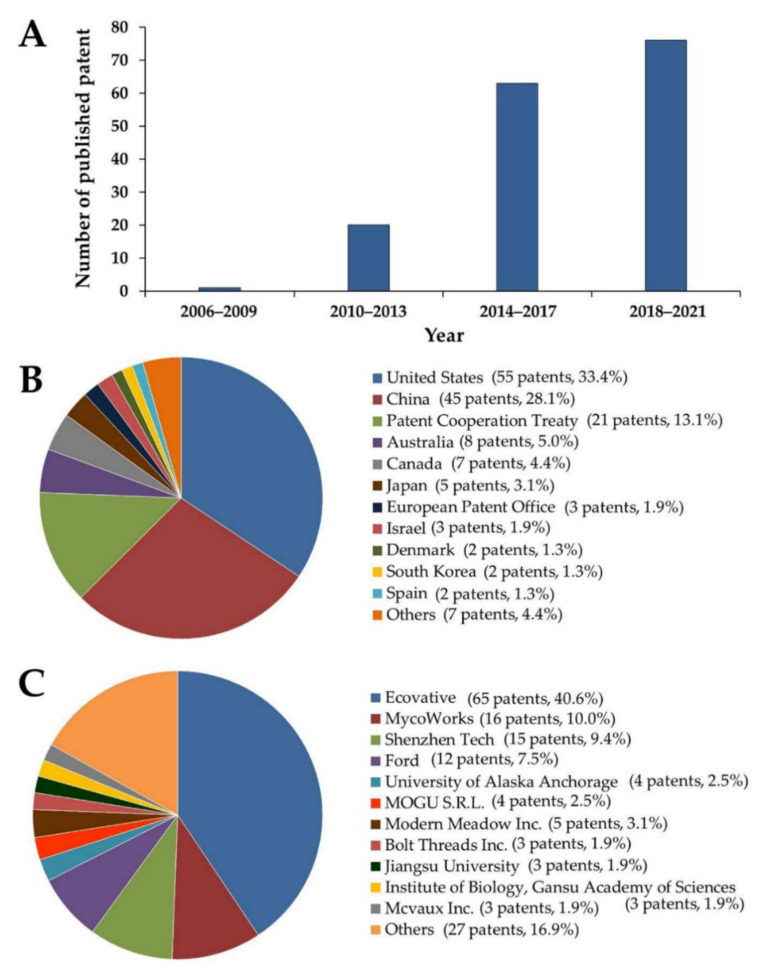
Number of patent (**A**), publication place (**B**) and patent ownership (**C**) between 2006 and 2021 of mycelium-based composite and related field. The search was performed using European database Espacenet and Google Patents (accessed on the 30 May 2022).

A patent search was carried out using the European database Espacenet [149] with additional validation using Google Patents [150]. A database search for “composite”, “fungi”, “leather”, “mycelium”, “material”, and “mycological” as keywords for patents retrieved 160 results for published patents during the time period from 2006 to 2021 (Supplementary Table S1). Patents in this field are widespread throughout the world and their applications have significantly increased since 2006 (Figure 6A). It was found that the majority of patents are owned by companies and universities. The majority of patents were published in the USA with 55 patents, followed by China with 45 patents, the Patent Cooperation Treaty (PCT) with 45 patents, Australia with eight patents, Canada with seven patents, and Japan with five patents (Figure 6B). The company Ecovative Design LLC (Ecovative) leads the way in patent publication with 40.6%, followed MycoWorks Inc., San Francisco, CA, USA, (MycoWorks) with 10.0%, Shenzhen Zeqingyuan Technology Development Service Co. Ltd., Shenzhen, China, (Shenzhen Tech) with 9.4%, Ford Global Tech (Ford) with 7.5%, and Modern Meadow Inc. with 4.1% (Figure 6C). The increasing trend in the registration and publication of applications for fungi to be used in functional materials is expected to continue in the future.

## 10. Conclusions and Future Perspectives

The search for biomaterials that can be used to replace plastic and facilitate the recycling of agricultural waste is currently of considerable interest. Over the last two decades, bio-fabrication technology that involves growing fungi on agricultural wastes has been developed to generate MBC. This paper has summarized the crucial details associated with this process, including fungal species, type of substrate, and the effects of each parameter on the properties of these MBC in terms of their potential applications. MBC are expected to become more popular as alternative materials in packaging, fashion, and architecture in the future. MBM have several key advantages over traditional synthetic materials, including their low cost, safety, and biodegradability, as well as for their relatively low environmental effects. However, the problem of low mechanical properties, high water absorption, and the absence of a standard set of methods for the manufacturing and testing of MBM remain major challenges that must be addressed in the future. An expanded set of parameters for the various substrate components must be developed and applied in order to understand their interactions and their impacts on the quality of the final materials. Additionally, more refined analytical methods should be employed to better evaluate the suitability of the MBM for each specific application.

## Figures and Tables

**Table 1:** The density of mycelium-based composites.

Fungal Species	Substrates	Pressing	Density (kg/m^3^)	Reference
*Agaricus bisporus*	Oat husk	–	36.0	[28]
	Rapeseed cake	–	58.0	[28]
*Coriolus* sp.	Apple wood chip	–	210.0	[24]
	Vine wood chip	–	180.0	[24]
*Ganoderma* sp.	Apple wood chip	–	220.0	[24]
	Vine wood chip	–	210.0	[24]
*Ganoderma lucidum*	Beech sawdust	Cold	205.3	[34]
	Chinese albizia sawdust	–	130.0	[35]
	Chinese albizia sawdust	Heat	954.0	[35]
	Oat husk	–	25.0	[28]
	Rapeseed cake	–	41.0	[28]
	Spent mushroom	Cold	183.2	[34]
*Ganoderma resinaceum*	Beechwood sawdust	–	143.0	[40]
	Lavender straw	–	347.0	[41]
	Miscanthus fiber	–	200.0	[39]
	Rose flowers	–	462.0	[41]
*Irpex lacteus*	Wood pulp	–	265.0	[47]
*Lentinus velutinus*	Pine sawdust	–	350.0	[51]
*Oudemansiella radicata*	Cotton stalk	–	317.0	[52]
*Pleurotus* sp.	Wheat straw	–	183.8	[48]
*Pleutorus albidus*	Pine sawdust	–	300.0	[51]
*Pleurotus ostreatus*	Cotton	–	130.0	[23]
	Cotton	Cold	240.0	[23]
	Cotton	Heat	350.0	[23]
	Cotton stalk	–	325.0	[52]
	Oat husk	–	38.0	[28]
	Pine wood shaving	Heat	290.0	[55]
	Rapeseed cake	–	49.0	[28]
	Rapeseed straw	–	130.0	[23]
	Rapeseed straw	Cold	240.0	[23]
	Rapeseed straw	Heat	390.0	[23]
	Rice husk	–	437.0	[57]
	Sawdust	–	178.5–552.0	[26,56,58]
	Sugarcane bagasse	–	110.0	[58]
	Straw	–	277.0	[26]
*Pycnoporus sanguineus*	Coconut powder	–	240.0	[64]
	Pine sawdust	–	320.0	[51]
*Trametes* sp.	Apple wood chip	–	200.0	[24]
	Vine wood chip	–	210.0	[24]
*Trametes hirsuta*	Pine wood shaving	Heat	260.0	[55]
*Trametes multicolor*	Beech sawdust	–	170.0	[23]
	Rapeseed straw	–	100.0	[23]
	Rapeseed straw	Heat	350.0	[23]
*Trametes versicolor*	Beech sawdust	Cold	200.1	[34]
	Flax	Cold	137.5	[25]
	Hardwood chips	–	179.0	[62]
	Hemp hurds	Cold	98.4	[25]
	Hemp shives	–	134.0	[62]
	Rice hull	–	193.0	[61]
	Spent mushroom	Cold	195.2	[34]
	Wheat straw	Cold	122.1	[25]

“–” = none pressing.

**Table 2:** The water absorption performance of mycelium-based composites.

Fungal Species	Substrates	Time (hours)	Value (%)	Standard Test	Reference
*Coriolus* sp.	Apple wood chip	96	240.0	ASTM C272	[24]
	Vine wood chip	96	290.0	ASTM C272	[24]
*Ganoderma* sp.	Apple wood chip	96	200.0	ASTM C272	[24]
	Vine wood chip	96	180.0	ASTM C272	[24]
*Ganoderma resinaceum*	Lavender straw	24	114.6	ISO 16535:2019	[41]
	Miscanthus fibers	22	125.0	Not mentioned	[39]
	Rose flowers	24	43.9	ISO 16535:2019	[41]
*Lentinula edodes*	Peach palm sheath	48	351.0	ASTM D570-98	[49]
*Oudemansiella radicata*	Cotton stalk	168	162.4	ASTM C272	[52]
*Pleurotus* sp.	Wheat straw	24	268.4	ASTM D570-98	[48]
*Pleurotus ostreatus*	Cotton	192	508.0	Not mentioned	[23]
	Cotton stalk	168	168.1	ASTM C272	[52]
	Hemp	96	159.0	Not mentioned	[53]
	Lacquer wood chip	96	135.0	Not mentioned	[53]
	Oak wood chip	96	76.0	Not mentioned	[53]
	Pine wood shaving	48	200.0	Not mentioned	[55]
	Rapeseed straw	192	279.0	Not mentioned	[23]
	Rice straw	96	140.0	Not mentioned	[53]
	Sawdust	24	131.0	ASTM D570-98	[58]
	Sugarcane bagasse	24	148.0	ASTM D570-98	[58]
*Trametes* sp.	Apple wood chip	96	200.0	ASTM C272	[24]
	Vine wood chip	96	190.0	ASTM C272	[24]
*Trametes hirsuta*	Pine wood shaving	48	200.0	Not mentioned	[55]
*Trametes multicolor*	Beech sawdust	192	43.0	Not mentioned	[23]
	Rapeseed straw	192	436.0	Not mentioned	[23]
*Trametes versicolor*	Flax	24	30.3	ASTM C1585	[25]
	Hardwood chip	24	400.0	ASTM D1037	[62]
	Hemp hurds	24	24.4	ASTM C1585	[25]
	Hemp shives	24	560.0	ASTM D1037	[62]
	Wheat straw	24	26.8	ASTM C1585	[25]

**Table 3:** Thermal properties of mycelium-based composites.

Thermal Properties	Fungal Species	Substrates	Value	Standard Test	Reference
Thermal conductivity (W/m∙K)	*Ganoderma lucidum*	Beech sawdust	0.070	Not mentioned	[34]
		Spent mushroom	0.064	Not mentioned	[34]
		Wheat straw	0.029	Not mentioned	[37]
	*Ganoderma resinaceum*	Miscanthus fibers	0.104	ISO 8302	[39]
		Wheat straw	0.081	Not mentioned	[22]
	*Irpex lacteus*	Wood pulp	0.070	ASTM D5334	[47]
	*Megasporoporia minor*	Wheat straw	0.079	Not mentioned	[22]
	*Oxyporus latermarginatus*	Wheat straw	0.078	Not mentioned	[22]
	*Pleurotus ostreatus*	Reed	0.070	Not mentioned	[56]
		Tomato stem	0.060	Not mentioned	[56]
	*Trametes versicolor*	Beech sawdust	0.067	Not mentioned	[34]
		Flax	0.059	ASTM D5334	[25]
		Hemp hurds	0.040	ASTM D5334	[25]
		Spent mushroom	0.064	Not mentioned	[34]
		Wheat straw	0.042	ASTM D5334	[25]
Thermal degradation (about 70% weight loss) (°C)	*Lentinus velutinus*	Pine sawdust	360	Not mentioned	[51]
	*Oudemansiella radicata*	Cotton stalk	310	Not mentioned	[52]
	*Pleurotus albidus*	Pine sawdust	355	Not mentioned	[51]
	*Pleurotus ostreatus*	Cotton	242	Not mentioned	[23]
		Cotton stalk	310	Not mentioned	[52]
		Rapeseed straw	225	Not mentioned	[23]
		Rubber sawdust	350	Not mentioned	[54]
		Sawdust	280	ASTM D3418	[59]
	*Pycnoporus sanguineus*	Pine sawdust	362	Not mentioned	[51]
	*Trametes multicolor*	Rapeseed straw	225	Not mentioned	[23]
	*Trametes versicolor*	Rice hull	250	Not mentioned	[61]
		Wheat grain	375	Not mentioned	[67]

**Table 4:** Mechanical properties of mycelium-based composites.

Mechanical Properties	Fungal Species	Substrates	Pressing	Value (MPa)	Standard Test	Reference
Compression strength	*Agaricus bisporus*	Oat husk	–	0.06	Not mentioned	[28]
		Rapeseed cake	–	0.20	Not mentioned	[28]
	*Fomes fomentarius*	Hemp shives	–	0.20	Not mentioned	[32]
		Rapeseed straw	–	0.30	Not mentioned	[32]
	*Ganoderma lucidum*	Chinese albizia sawdust	Heat	4.44	ASTM D1037	[35]
		Oat husk	–	0.13	Not mentioned	[28]
		Rapeseed cake	–	0.28	Not mentioned	[28]
		Red oak chips	–	0.49	ASTM D3574	[38]
		Wheat straw	–	0.07	ISO 844	[37]
	*Ganoderma resinaceum*	Beech sawdust	–	1.32	ISO EN 826	[40]
		Lavender straw	–	0.72	ISO EN 826	[41]
		Miscanthus fibers	–	1.80	ISO 844	[39]
		Rose flowers	–	1.03	ISO EN 826	[41]
	*Irpex lacteus*	Wood pulp	–	0.57	ASTM D2166	[47]
	*Lentinula edodes*	Coconut powder	–	0.06	Not mentioned	[64]
		Peach palm sheath	–	0.22	ASTM 165	[49]
	*Lentinus velutinus*	Pine sawdust	–	1.30	Not mentioned	[51]
	*Oudemansiella radicata*	Cotton stalk	–	0.09	ASTM D2166	[52]
	*Pleurotus* sp.	Wheat straw	–	0.04	ASTM C165	[48]
	*Pleutorus albidus*	Pine sawdust	–	0.40	Not mentioned	[51]
	*Pleurotus ostreatus*	Cotton stalk	–	0.13	ASTM D2166	[52]
		Oat husk	–	0.03	Not mentioned	[28]
		Rapeseed cake	–	0.28	Not mentioned	[28]
		Rice husk	–	1.35	Not mentioned	[57]
		Sawdust	–	1.02	Not mentioned	[26]
		Straw	–	0.07	Not mentioned	[26]
	*Pycnoporus sanguineus*	Coconut powder	–	0.19	ASTM 1621	[64]
		Pine sawdust	–	1.30	Not mentioned	[51]
	*Trametes versicolor*	Flax	Cold	0.31	ASTM D5334	[25]
		Hemp hurds	Cold	0.51	ASTM D5334	[25]
		Pine wood	Cold	0.14	ASTM D5334	[25]
Tensile strength	*Ganoderma lucidum*	Chinese albizia sawdust	Heat	1.55	ASTM D1037	[35]
		Red oak chips	–	0.18	ASTM D3574	[38]
	*Pleurotus* sp.	Wheat straw	–	0.05	ASTM D1623	[48]
	*Pleurotus ostreatus*	Cotton	Cold	0.03	Not mentioned	[23]
		Cotton	Heat	0.13	Not mentioned	[23]
		Rapeseed straw	–	0.01	Not mentioned	[23]
		Rapeseed straw	Cold	0.03	Not mentioned	[23]
		Rapeseed straw	Heat	0.24	Not mentioned	[23]
	*Trametes multicolor*	Beech sawdust	–	0.05	Not mentioned	[23]
		Rapeseed straw	–	0.04	Not mentioned	[23]
		Rapeseed straw	Heat	0.15	Not mentioned	[23]
Flexural strength	*Ganoderma lucidum*	Chinese albizia sawdust	Heat	2.68	ASTM D1037	[35]
		Cotton stalk	Heat	4.40	GB/T 17657	[36]
	*Pleurotus ostreatus*	Cotton	–	0.05	Not mentioned	[23]
		Cotton	Cold	0.24	Not mentioned	[23]
		Cotton	Heat	0.62	Not mentioned	[23]
		Pine wood shaving	Heat	0.94	ASTM D7264	[55]
		Rapeseed straw	–	0.06	Not mentioned	[23]
		Rapeseed straw	Cold	0.21	Not mentioned	[23]
		Rapeseed straw	Heat	0.87	Not mentioned	[23]
		Rubber sawdust	Heat	3.91	JIS A5908	[54]
	*Trametes hirsuta*	Pine wood shaving	Heat	0.94	ASTM D7264	[55]
	*Trametes multicolor*	Beech sawdust	–	0.29	Not mentioned	[23]
		Rapeseed straw	–	0.22	Not mentioned	[23]
		Rapeseed straw	Heat	0.86	Not mentioned	[23]

“–” = none pressing.

**Table 5:** Final pH value and nitrogen content of mycelium-based composites.

Fungal Species	Substrates	Final pH Value	Nitrogen Content (%)	Reference
*Coriolus* sp.	Apple wood chip	4.5	Not determined	[24]
	Vine wood chip	4.5	Not determined	[24]
*Cyclocybe aegerita*	Apple wood chip	5.8	0.8	[30]
	Eucalyptus wood chip	6.5	0.9	[30]
	Oak wood chip	6.0	0.6	[30]
	Pine wood chip	6.3	0.6	[30]
*Ganoderma* sp.	Apple wood chip	4.5	Not determined	[24]
	Vine wood chip	4.5	Not determined	[24]
*Lentinula edodes*	Peach palm sheath	6.0	1.1	[49]
*Pleurotus ostreatus*	Apple wood chip	4.6	0.7	[30]
	Eucalyptus wood chip	4.3	1.1	[30]
	Oak wood chip	4.8	0.8	[30]
	Pine wood chip	4.3	0.5	[30]
	Vine wood chip	4.7	1.0	[30]
*Pleurotus pulmonarius*	Apple wood chip	5.3	0.7	[30]
	Eucalyptus wood chip	5.2	0.9	[30]
	Oak wood chip	5.5	0.8	[30]
	Pine wood chip	5.4	0.6	[30]
	Vine wood chip	5.5	1.1	[30]
*Pleurotus salmoneostramineus*	Eucalyptus wood chip	4.7	0.8	[30]
	Oak wood chip	5.2	0.8	[30]
	Pine wood chip	4.7	0.7	[30]
*Trametes* sp.	Apple wood chip	4.5	Not determined	[24]
	Vine wood chip	4.5	Not determined	[24]
*Trametes versicolor*	Hardwood chip	None	0.7	[66]
	Hemp shives	None	1.6	[66]

**Table 7:** Comparison of mycelium architecture review project (modified from Almpani-Lekka et al. [129]).

Project/Year of Completion	Location	Type	Structure	Fungus	Substrate	Post-Treatment	Creator
HY-FI (2014)	Outside	Brick	Wood and Steel	*Ganoderma* *lucidum*	Corn stalks	Heat treated	The Living Studio
Shell mycelium (2017)	Outside	Panel	Wood and Steel	Not mentioned	Coir pith	Naturally dried	Studio Beetles 3.3 Yassin Arredia Design
Mycotree (2017)	Inside	Block	Bamboo and Steel	*Pleurotus* *ostreatus*	Sugar cane, Cassava root	Heat treated	Sustainable Construction KIT Karlsruhe Block Research Group ETH Zurich
Monolito Micelio (2020)	Outside	Monolith	Wood and Steel	*Ganoderma* *lucidum*	Hemp	Naturally dried	Georgia Institute of Technology School of Architecture
Growing Pavilion (2020)	Outside	Panel	Wood	*Ganoderma lingzhi*	Hemp, Cattail, and Mace	Heat treated and Weather resistant biocoating	Company New Heroes E. Klarenbeek
My-Co Space (2021)	Outside	Panel	Wood and Steel	*Fomes* *fomentarius*	Hemp	Heat treated and Weather resistant biocoating	MY-CO-X Collective

## Data Availability

Not applicable.

## Supplementary Materials

The following supporting information can be downloaded at: https://www.mdpi.com/article/10.3390/jof8080842/s1, Table S1: List of patents, publication place and patent ownership between 2006 and 2021 of mycelium-based composite and related field.
